# Malignancy Risk and Predictors in Dermatomyositis and Polymyositis: A Large Population-Based Study

**DOI:** 10.3390/medicina61111932

**Published:** 2025-10-28

**Authors:** Yonatan Shneor Patt, Niv Ben-Shabat, Paula David, Chen Patt, Kassem Sharif, Yoav Elizur, Ido Cohen, Arnon D. Cohen, Howard Amital, Abdulla Watad, Omer Gendelman

**Affiliations:** 1 Department of Internal Medicine B & Zabludowicz Center for Autoimmune Diseases, Sheba Medical Center, Tel-Hashomer, Ramat Gan 5262100, Israel; yopatt123@gmail.com (Y.S.P.); nivben7@gmail.com (N.B.-S.); paulardavid@gmail.com (P.D.); yoav.elizur@mail.huji.ac.il (Y.E.); howard.amital@sheba.health.gov.il (H.A.); omer.g79@gmail.com (O.G.); 2 Gray Faculty of Medical and Health Sciences, Tel-Aviv University, Tel-Aviv 6927846, Israel; chenktz@gmail.com (C.P.); kassemsharif@gmail.com (K.S.); idocomail@gmail.com (I.C.); 3 Dina Recanati School of Medicine, Reichman University, Herzliya 4610101, Israel; 4 Section of Musculoskeletal Disease, NIHR Leeds Musculoskeletal Biomedical Research Unit, Chapel Allerton, Leeds Teaching Hospital Trust, Leeds LS1 3EX, UK; 5 Center for Advanced Endoscopy, Harvard Medical School, Boston, MA 02108, USA; 6 Department of Internal Medicine E, Sheba Medical Center, Tel-Hashomer, Ramat Gan 5262100, Israel; 7 Chief Physician’s Office, Clalit Health Services, Tel-Aviv 6209813, Israel; arcohen@clalit.org.il; 8 Siaal Research Center for Family Medicine and Primary Care, Faculty of Health Sciences, Ben-Gurion University of the Negev, Beer-Sheva 8410501, Israel; 9 Rheumatology Unit, Sheba Medical Centre, Tel-Hashomer, Ramat Gan 5262100, Israel

**Keywords:** dermatomyositis, polymyositis, malignancy, cancer, epidemiology, autoantibodies

## Abstract

*Background and Objectives*: Idiopathic inflammatory myopathies (IIMs) are rare autoimmune diseases with extra-muscular manifestations. A firm association with malignancy, mainly observed in dermatomyositis (DM) is well established and several predictors of malignancy were previously published. However, given the low prevalence of IIMs, large population-based studies are scarce, hindering a comprehensive understanding of site-specific cancer patterns and risk factors. This study aimed to evaluate malignancy patterns and predictors, in a large, diverse cohort of patients with DM and PM. *Materials and Methods*: This retrospective cohort study used the Clalit Health Services electronic database, including 1557 DM patients and 528 PM patients diagnosed between 2002 and 2018, along with age-, sex-, and residence-matched controls at a 1:5 ratio. The incidence and risk of malignancies were assessed using Cox proportional hazards models, and predictors of solid and hematologic malignancies within the PM/DM cohort were analyzed using adjusted logistic regression. *Results:* In our cohort, DM was associated with an increased risk of both solid and hematologic malignancies (HR 1.89, 95%CI 1.47–2.41), whereas in PM the association was less pronounced and limited to solid malignancies (HR 1.50, 95%CI 1.06–2.11). In DM, higher risks were observed for breast cancer (HR 1.86) and chronic leukemia (HR 5.02). Across both subtypes, older age at diagnosis, the presence of specific autoantibodies were associated with increased malignancy risk. Significant markers included antiphospholipid antibodies (OR 2.28), lupus anticoagulant (OR 2.29), anti-Mi2 (OR 2.09), any of the antinuclear antibodies (OR 2.37), and individually anti-RNP/Sm (OR 1.70), anti-Ro/La (OR 1.79), anti-Scl-70 (OR 1.60), and anti-DNA (OR 1.97). *Conclusions:* Our study demonstrates an increased risk of malignancy in both DM and PM, with the relationship being stronger and broader in DM, involving both solid and hematologic cancers. Older age at diagnosis, and a distinct serological profile, particularly antiphospholipid antibodies and various antinuclear antibodies, identify patients at highest risk, warranting heightened clinical vigilance.

## 1. Introduction

Idiopathic inflammatory myopathies (IIMs) are a group of rare, heterogeneous, multisystem autoimmune diseases primarily characterized by inflammation of striated muscle. The two major subtypes, dermatomyositis (DM) and polymyositis (PM), typically present with proximal muscle weakness, fatigue, and myalgia [1].

Beyond their clinical similarities, PM and DM also share considerable immunopathological features [2,3]. Histologically, both subtypes demonstrate muscle fiber degeneration and regeneration, as well as microvascular injury and capillary depletion. At the molecular level, overexpression of proinflammatory cytokines and chemokines—such as interferon-gamma (IFN-γ); interferon-alpha (IFN-α); interleukins (IL)-1, IL-2, IL-17, and IL-18; and tumor necrosis factor-alpha (TNF-α)—has been observed in both conditions [4,5]. Inflammatory cell infiltration is also common to both, with dendritic cells, macrophages, eosinophils, and natural killer (NK) cells contributing to the local immune response. However, notable differences in T-cell profiles exist—PM is characterized by endomysial infiltration of CD8+ T lymphocytes, while DM typically exhibits perimysial CD4+ T-cell infiltration [3,6].

Extra-muscular manifestations are frequently observed in IIMs and may include interstitial lung disease, arthritis, and calcinosis. Distinctive cutaneous features such as heliotrope rash and Gottron’s papules are hallmark findings in DM but are absent in PM [7,8]. Importantly, a well-established association exists between IIMs and malignancy, which is considered a leading cause of mortality among affected patients [9,10]. The evidence is particularly robust for DM whereas data specific to PM remain limited and often less significant, partly due to the frequent combining of PM and DM in analyses, which obscures PM-specific risk estimates [11,12,13,14]. A previous systematic review and meta-analysis by Qiang et al. [15] reported an overall relative risk of malignancy of 4.66 in DM and 1.75 in PM, underscoring the heightened cancer burden in this population and its adverse prognostic implications.

In this context, a variety of different autoantibodies are detected in approximately 50–70% of patients with IIMs. Although their precise pathogenic role remains incompletely understood, these serological markers have proven valuable in clinical practice. Autoantibody profiles are associated with distinct clinical phenotypes, particularly patterns of organ involvement and malignancy risk [16,17].

Given the rarity of these diseases, most available studies are constrained by relatively small sample sizes and frequently rely on meta-analyses to increase statistical power [18,19,20]. Consequently, limited information exists regarding malignancy predictors within the IIM population, including the influence of demographic and clinical characteristics and distinct autoantibody profiles.

To address these knowledge gaps, we utilized a large, population-based cohort from Clalit Health Services (CHS), the largest health maintenance organization (HMO) in Israel, to examine the association between myositis and malignancy in a diverse, real-world setting. Through this comprehensive analysis, we aimed to (i) determine the overall risk of malignancy in patients with DM and PM, (ii) characterize site-specific malignancy patterns, and (iii) identify subgroups at highest risk. These objectives are intended to inform strategies for tailored malignancy screening and surveillance in this vulnerable population.

## 2. Materials and Methods

### 2.1. Data Source

This study received approval from the Clalit Health Services (CHS) Ethics Committee in Tel Aviv, Israel (approval number: 0212-17-COM; approval date: 3 January 2018, with extension until 15 April 2026). Given that this was a database-based study utilizing existing electronic medical records, the requirement for informed consent was waived.

Data were obtained from the Clalit Healthcare Services (CHS) comprehensive electronic database, which is the largest health maintenance organization in Israel, serving approximately 4.5 million insured members (over 50% of the Israeli population) from heterogeneous ethnic backgrounds. The database continuously receives real-time input from pharmaceutical, medical, and administrative systems and is designed for administrative and clinical management, as well as epidemiological research. Diagnoses undergo a continuous validation process through logistic checks (such as cross-referencing diagnoses from different sources), and previous studies have demonstrated the high reliability of the CHS database [21,22].

### 2.2. Study Population and Design

This study was designed as a retrospective cohort study. Using the CHS computerized database, we identified all individuals diagnosed with PM or DM (ICD-9 codes 710.4 and 710.3, respectively) between 1 January 2002 and 31 December 2018. These patients were compared to a control group of individuals without a PM/DM diagnosis, matched 1:5 by age, sex, and place of residence.

Follow-up for each participant began at the date of PM/DM diagnosis (or matched index date for controls) and continued until the earliest occurrence of malignancy, death, or end of study follow-up on 1 July 2019.

### 2.3. Study Variables

All variables were obtained from CHS electronic health records. Malignancy diagnosis was based on recorded medical documentation within the CHS database. A patient was considered to have malignancy if they had a documented diagnosis of any type of cancer. Malignancies were categorized based on whether they were diagnosed before or after the PM/DM diagnosis (or index date for controls), with only malignancies first diagnosed after the PM/DM diagnosis considered incident cases. Overall cancer was defined as having at least one malignancy, including both solid and hematologic malignancies.

Malignancies were further classified into:

- Solid malignancies: Central nervous system (CNS), oropharyngeal, laryngeal, thyroid, lung, breast, uterine, ovarian, cervical, genital, kidney, bladder, prostate, esophageal, gastric, colorectal, liver, biliary, pancreatic, sarcoma, melanoma, and unknown primary.
- Hematologic malignancies: Acute leukemia, chronic leukemia, Hodgkin’s lymphoma, non-Hodgkin’s lymphoma, multiple myeloma and myelodysplastic syndrome.

Demographic variables included age, sex, ethnicity (categorized as Jewish or Arab), socioeconomic status (SES) (classified based on area of residence using data from the Israeli National Census), body mass index (BMI) at the nearest time to diagnosis, and smoking status (ever vs. never).

Laboratory values included C-reactive protein (CRP) and creatine phosphokinase (CPK), recorded as the highest values measured post-PM/DM diagnosis (or matched date for controls). Serology tests for autoantibodies were also retrieved, including anti-Jo1, anti-Mi2, antinuclear antibody (ANA), anti-ribonucleoprotein (anti-RNP), anti-Smith (Sm), anti-Ro/SSA, anti-La/SSB, anti-Scl-70, anti-DNA, anti-beta-2-glycoprotein (B2GP), anti-cardiolipin (ACL), and lupus anticoagulant (LAC). Anti-RNP with anti-Sm and anti-Ro with anti-La were grouped together accordingly. A test was considered positive if classified as such by the performing laboratory, based on its predefined cutoff. All laboratory tests were conducted in the CHS central laboratory during the follow-up period.

### 2.4. Statistical Analysis

Continuous variables were presented as mean ± standard deviation (SD) and compared using Student’s *t*-test. Categorical variables were reported as percentages and compared using the Pearson’s chi-square test of independence; when expected cell counts were too small, Fisher’s exact test was applied. Differences were considered statistically significant at *p* < 0.05.

The incidence and risk of malignancies among PM/DM patients compared to controls were evaluated using Cox proportional hazards regression models, including only incident cases. Additionally, risk factors for solid and hematologic malignancies within the PM/DM cohort were analyzed using age- and sex-adjusted logistic regression models.

All statistical analyses were performed using SPSS software (version 26, IBM Corp., Armonk, NY, USA).

## 3. Results

### 3.1. Study Population

The study included 528 patients with PM and 1557 patients with DM, along with age-, sex-, and residence-matched control groups. The mean age at diagnosis was comparable between cases and controls in both PM and DM. Females constituted the majority in both disease groups, with similar proportions across cases and controls (63% in PM and 57.5% in DM). There were no significant differences in socioeconomic status or ethnicity between patients with PM/DM and their corresponding controls. BMI was slightly lower in DM patients compared to controls (*p* = 0.05, not statistically significant), and no significant differences in BMI were observed in PM patients.

### 3.2. Overall Cancer Rates and Timing Relative to PM/DM Diagnosis

A significantly higher proportion of patients with PM and DM had a cancer diagnosis compared to their respective controls (16.1% vs. 11.9%, *p* = 0.009; 10.2% vs. 7.0%, *p* < 0.001, respectively). When stratified by the timing of cancer diagnosis in relation to myositis diagnosis, the prevalence of cancer prior to the index date did not differ significantly between cases and controls for either PM or DM. However, diagnosis of cancer after myositis onset was significantly frequent among patients with PM (8.7% vs. 6.0%, *p* = 0.025) and DM (5.8% vs. 3.3%, *p* < 0.001) than in their respective controls. The mean age at cancer diagnosis prior to the index date did not differ significantly between cases and controls. However, among patients with PM and DM, cancers diagnosed after myositis onset occurred at a significantly younger age compared to controls (57.9 vs. 65.9 years, *p* = 0.004; 55.6 vs. 60.4 years, *p* = 0.011, respectively). For further details, see Table 1.

### 3.3. Site-Specific Malignancies

Patients with PM had a significantly increased overall risk of developing malignancy compared to controls, with an age- and sex-adjusted hazard ratio (HR) of 1.50 (95% CI: 1.06–2.11, *p* = 0.020). When analyzed by cancer type, the risk of solid malignancies was also significantly elevated in PM patients (HR = 1.50, 95% CI: 1.05–2.14, *p* = 0.025), whereas no significant difference was observed for hematologic malignancies (HR = 1.63, 95% CI: 0.53–5.07, *p* = 0.395).

Similarly, patients with DM had a significantly increased overall risk of developing malignancy compared to controls, with an age- and sex-adjusted HR of 1.89 (95% CI: 1.47–2.41, *p* < 0.001). The risk of solid malignancies was also significantly higher among DM patients (HR = 1.73, 95% CI: 1.33–2.25, *p* < 0.001). Hematologic malignancies were likewise more frequent in DM patients (HR = 3.26, 95% CI: 1.62–6.55, *p* < 0.001).

Regarding site-specific cancers, DM patients demonstrated a significantly increased risk of chronic leukemia (HR = 5.02, 95% CI: 1.62–15.56, *p* = 0.005), and breast cancer (HR = 1.86, 95% CI: 1.07–3.25, *p* = 0.028) with a trend toward increased risk for laryngeal cancer and Hodgkin’s lymphoma. For further details, see Table 2.

### 3.4. Risk Factors for Solid and Hematologic Malignancies in Patients with PM and DM

An analysis of cancer predictors within the PM and DM cohort identified several significant factors influencing the risk of developing solid malignancies. Older age at diagnosis was significantly associated with a higher risk of solid malignancy, with an OR of 1.14 per 5-year increment. Other demographic variables, including obesity, female sex, Arab ethnicity, and low socioeconomic status, were not significantly associated with solid malignancy risk. Inflammatory markers, including CPK levels and CRP levels were not associated with solid malignancies as well.

When assessing the impact of autoantibodies on malignancy risk, several markers were found to significantly increase the likelihood of developing solid cancers. Among myositis-specific autoantibodies, the presence of Anti-Mi2 was significantly associated with an elevated risk (OR = 2.09, 95% CI: 1.22–3.58, *p* = 0.007), whereas Anti-Jo1 did not show a significant association. The presence of any anti-nuclear antibody was associated with a significantly increased risk of solid malignancies (OR = 2.37, 95% CI: 1.44–3.91, *p* < 0.001). This association remained significant also when the different anti-nuclear antibodies were analyzed individually, including Anti-RNP/Sm, Anti-Ro/La, Anti-Scl-70, and Anti-DNA. Regarding the antiphospholipid antibodies (APLA), anti-B2GP, ACL and lupus anticoagulant (LAC) were all significantly associated with an increased risk of solid malignancy (OR = 2.28, 95% CI: 1.55–3.35, *p* < 0.001). The risk appeared to remain significant with both double positivity for APLA (ACL and B2GP1) and triple positivity APLA (LAC).

Regarding hematologic malignancies, as with solid cancers, older age at diagnosis was significantly associated with an increased risk with an OR of 1.14 per 5-year increment (95% CI: 1.01–1.28, *p* = 0.032). Other demographic factors as well as inflammation and CPK were not significantly associated with hematologic malignancy risk.

An analysis of the different autoantibodies and their association with hematologic malignancies revealed several significant findings. Among myositis-specific autoantibodies, both Anti-Jo1 and Anti-Mi2 were significantly associated with an increased risk of hematologic malignancies. A similar trend was observed for Anti-Ro/La (OR = 3.02, 95% CI: 1.06–8.57, *p* = 0.038) and Anti-DNA (OR = 5.31, 95% CI: 1.44–19.55, *p* = 0.012). Neither APLA nor LAC demonstrated a significant association with hematologic malignancy risk. For more information, see Table 3.

## 4. Discussion

In this large, population-based cohort study, we found that both DM and PM were associated with a significantly higher prevalence of malignancy compared to matched controls (DM: 10.2% vs. 7.0%, *p* < 0.001; PM: 16.1% vs. 11.9%, *p* = 0.009). In the majority of cases, malignancies were diagnosed after the onset of myositis and occurred at a younger age compared to the controls. Although the risk of developing malignancy was elevated in both DM and PM, the association was more pronounced among DM patients, encompassing both solid and hematologic malignancies (HR 1.89, 95% CI 1.47–2.41, *p* < 0.001), whereas in PM patients the increased risk was limited to solid malignancies alone (HR 1.50, 95% CI 1.06–2.11, *p* = 0.020). Regarding site-specific cancers, DM patients had a significantly increased risk of breast cancer (HR 1.86, 95% CI 1.07–3.25) and chronic leukemia (HR 5.02, 95% CI 1.62–15.56), with a trend toward increased risk for laryngeal cancer and Hodgkin’s lymphoma. We have found several risk factors for developing malignancy, including older age at diagnosis and specific autoantibodies, such as anti-Mi2, different antinuclear antibodies, and antiphospholipid antibodies.

Previous studies extensively examined the association between IIMs and malignancy. Whereas in our cohort, the HR was 1.89 for DM and 1.50 for PM, several earlier studies reported higher magnitudes of risk. For instance, Buchbinder et al. [13] observed standardized incidence ratio (SIRs) of 6.2 in DM and 2.0 in PM. Conversely, a meta-analysis of 69 studies reported a pooled risk ratio of 2.2 for malignancy in DM patients, which is more consistent with our findings [10]. However, the risk in PM remains less clear, with some large-scale studies reporting elevated risk in both subtypes, while others observed no significant increase in PM [10,14,23]. A retrospective study based on a Northern New England cohort, for example, reported a clear association between DM and malignancy, with SIRs of 5.57 (95% CI 2.97–9.53) in females and 3.43 (95% CI 0.94–8.79) in males, whereas no association was found for PM patients [24]. In contrast, a large population-based study of 537 patients with inflammatory myopathies demonstrated an elevated risk in both DM and PM, although the relative risk of malignancy was 2.4 times higher in DM compared to PM [13]. Taken together, most studies, even those reporting an increased risk among PM patients, have demonstrated a substantially greater difference in malignancy risk between DM and PM than observed in our cohort.

It is well established that most malignancies associated with IIMs are solid tumors, mainly adenocarcinomas, yet hematological malignancies were seldom reported. Indeed, a meta-analysis of 20 studies by Yang et al. [20], found that while DM was significantly associated with various solid tumors, particularly ovarian and lung cancers, no increased risk of lymphoma or Hodgkin disease was observed. In contrast, a retrospective study of PM and DM patients demonstrated a clear occurrence of hematologic malignancies, most commonly lymphomas [18]. These findings align with our results, in which DM was associated with both solid and hematologic malignancies emphasizing the need for screening for such malignancies in patients with inflammatory myopathies [25].

Regarding site-specific cancers, in our cohort, a significantly increased risk was observed only for breast cancer, with a non-significant trend toward laryngeal cancer, differing from prior literature that typically emphasizes also the involvement of the ovary and lung [23,26]. Such differences may be explained by genetic and environmental factors, as suggested by geographical variation in reported associations. In several Asian countries, such as South Korea, Singapore, southeastern China, Hong Kong and Taiwan, DM has been more frequently associated with breast, stomach, and nasopharyngeal cancers, whereas studies from Northern Europe have more commonly reported associations with ovarian and lung cancers [27,28]. In Israel, where the population is characterized by substantial genetic diversity, epidemiological data on IIM-associated malignancies are limited; however, existing studies suggest elevated risks for breast cancer, hematologic malignancies, and nasopharyngeal carcinoma [29,30]. These previously reported associations are consistent with our findings, which are based on a substantially larger Israeli cohort. Nevertheless, it is important to note that the numbers of laryngeal cancer and Hodgkin lymphoma cases in our cohort were very small, and these observations should therefore be regarded as hypothesis-generating only.

Serologically, several autoantibodies have been consistently linked to cancer-associated myositis, most notably anti-TIF1-γ, anti-NXP2, and anti-SAE. These antibodies are more frequently detected in patients with DM who develop malignancy, particularly within the first year after diagnosis. While their pathogenic role remains uncertain, they may represent a response to tumor-derived antigens or mutated cellular proteins, functioning as biomarkers rather than direct mediators. Some studies have shown declining antibody titers following cancer remission, further supporting their role as markers of tumor burden rather than causative agents [31,32].

In our cohort, several autoantibody profiles were significantly associated with an increased likelihood of solid malignancy, independent of age and sex. Among myositis-specific antibodies, anti-Mi2 was associated with approximately a twofold increase in solid cancer odds, whereas anti-Jo1 did not show a statistically significant elevated risk.

The evidence from previous research regarding a meaningful elevation in cancer risk for patients with anti-Mi2 is mixed [33]. Large tertiary-center and longitudinal investigations that examined cancer risk by specific myositis-related antibodies have repeatedly identified anti-TIF1-γ, and in some reports anti-NXP2 or anti-SAE, as the principal cancer-associated MSAs. In contrast, anti-Mi2 and anti-Jo1 usually represent only a small proportion of cancers occurring close to myositis onset and rarely show statistically significant standardized incidence ratios compared with the general population [34,35]. Nevertheless, Betteridge et al. [36] found that anti-Mi-2 autoantibodies were associated with an increased cancer risk, with an odds ratio of 2.5 for cancer-associated myositis, which is similar to the findings observed in our cohort.

Interestingly, in our cohort, both anti-Jo1 and anti-Mi2 were significantly associated with hematologic malignancies, with odds ratios of 4.31 (95% CI 1.45–12.80, *p* = 0.009) and 3.83 (95% CI 1.22–12.03, *p* = 0.021), respectively. This is notable, as anti-Jo1 has not been consistently linked to an increased risk of solid cancers in most previous studies. However, because the number of hematologic cancers was small (n = 17), these associations should be interpreted with caution and regarded as hypothesis-generating rather than definitive, as limited case numbers may bias the estimated predictive effects. In the existing literature, malignancies in IIM are typically reported as a combined outcome, without distinguishing between hematologic and solid tumors, leaving the relationship between specific myositis-specific antibodies and hematologic malignancies largely unexplored. A few case reports have described anti-Jo1-positive myositis occurring in conjunction with Hodgkin lymphoma [37]. However, in contrast to our findings, a retrospective series of 32 DM/PM patients with hematologic malignancies found that anti-Jo1 was significantly less common in affected individuals (*p* = 0.001), suggesting a possible protective association [18].

In our study, the presence of multiple anti-nuclear antibodies was significantly associated with increased odds of solid cancer. Having any of the tested antibodies (anti-RNP/Sm, anti-Ro/La, anti-Scl-70, or anti-DNA) was associated with more than a twofold increased risk (OR 2.37, 95% CI 1.44–3.91, *p* < 0.001). Individually, anti-RNP/Sm (OR 1.70, 95% CI 1.16–2.50, *p* = 0.007), anti-Ro/La (OR 1.79, 95% CI 1.22–2.64, *p* = 0.003), anti-Scl-70 (OR 1.60, 95% CI 1.29–1.99, *p* < 0.001), and anti-DNA (OR 1.97, 95% CI 1.31–2.99, *p* < 0.001) were each associated with significantly increased odds of cancer. To our knowledge, this is the first report describing such associations specifically in patients with DM. In contrast to our results, a retrospective cohort of 231 adult-onset DM patients found ANA-positive individuals had a markedly lower risk of malignancy within 3 years compared with ANA-negative patients (11% vs. 43%, OR 0.16) [38]. Nonetheless, beyond the IIM setting, studies in broader cancer populations have reported a higher prevalence of ANA in patients with malignancy compared with healthy controls, with ANA positivity linked to an increased frequency of musculoskeletal, paraneoplastic rheumatic manifestations, and poorer prognosis [39,40].

Outside the context of DM, prior studies have described links between these autoantibodies and cancer risk in other autoimmune populations. Anti-Ro/La antibodies, classically associated with Sjögren’s syndrome and SLE, have been linked to increased risks of melanoma, T-cell lymphoma, non-Hodgkin lymphoma, and breast carcinoma, particularly in older patients with systemic features such as fever, anemia, or cutaneous lupus erythematosus [41,42]. Isolated anti-Ro52 positivity has also been associated with malignancy in scleroderma and even in patients without overt autoimmune disease [43]. Furthermore, anti-dsDNA and extractable nuclear antigen antibodies in general (anti-ENA) have been associated with higher risk of diffuse large B-cell lymphoma and marginal zone lymphoma in prospective case–control cohorts [44]. Taken together, these observations across diverse autoimmune conditions provide external support for our findings, suggesting that certain autoantibody profiles may represent shared markers of increased cancer susceptibility.

The presence of antiphospholipid antibodies was also found to pose an increased risk for solid tumors, with double and triple positivity conferring the highest odds ratios, up to 2.70. To the best of our knowledge, this is the first time APLA and LAC are related to a higher risk of malignancy among DM/PM patients. Nevertheless, these findings align with prior literature showing that antiphospholipid antibodies, particularly ACL antibodies, are detected more frequently in patients with solid tumors than in the general population [45,46]. Reports consistently describe this pattern across gastrointestinal, genitourinary, and lung cancers, while associations for lupus anticoagulant and anti–β2 glycoprotein I are more variable [47,48]. Some studies also suggest a bidirectional relationship, where malignancy can trigger antiphospholipid formation and, conversely, established antiphospholipid syndrome may predispose to future malignancy [49]. Experimental evidence supports a mechanistic link, as antiphospholipid antibodies have been shown to promote tumor progression through tissue factor-dependent angiogenic pathways [50].

While our study offers notable strengths, including the use of a large, comprehensive, and well-established database, an extended follow-up period, and representation of an ethnically diverse population, several limitations should be acknowledged. First, the diagnoses of DM and PM were based exclusively on specialist-assigned ICD coding. In registry-based studies, diagnostic codes are the only available identifiers, and therefore the precise method by which each diagnosis was established (e.g., clinical evaluation, histopathological confirmation, or application of formal classification criteria) cannot be verified. Because our cohort included patients diagnosed between 2002 and 2018, most were not classified according to the 2017 ACR/EULAR criteria, and diagnostic practices likely varied over time [51]. This reliance on coding may therefore have introduced misclassification related both to evolving classification systems and to local differences in diagnostic approach. In addition, while comparing the patient group with a larger (1:5) control group increased statistical power and is supported by the epidemiologic methods literature [52], it may also increase the likelihood of detecting statistically significant differences, potentially introducing bias into the final results. Finally, while the database included a basic myositis antibody panel and several broader autoantibody assays, key cancer-associated antibodies, such as anti-TIF1-γ, anti-NXP2, and anti-SAE, were not available for analysis. The absence of these specific antibodies in IIM, particularly in the context of malignancy, further limits the interpretation of the data.

## 5. Conclusions

In conclusion, this large population-based study demonstrates a significant association between both DM and PM and the risk of malignancy, with a more pronounced relationship observed in DM, encompassing both solid and hematologic cancers. Within our cohort, several established predictors of malignancy were confirmed, including older age at IIM diagnosis and the presence of specific autoantibodies such as anti-Mi2. Notably, we also identified, for the first time, an association between malignancy and antiphospholipid antibodies, as well as anti-RNP/Sm, anti-Ro/La, anti-Scl-70, and anti-DNA antibodies. These findings highlight the need for heightened clinical vigilance in the management of IIM patients, particularly among those with high-risk serological profiles.

## Figures and Tables

**Table 1:** Baseline characteristics of the study population.

Characteristics	Polymyositis (*n* = 528)	Controls (*n* = 2560)	*p*	Dermatomyositis (*n* = 1557)	Controls (*n* = 7633)	*p*
	Demographics					
Age at diagnosis						
Mean ± SD	51.2 ± 18	50.9 ± 18	0.689	36.7 ± 22	36.4 ± 22	0.649
Median (IQR)	52.7 (38–65)	52.3 (38–65)	0.811	35.6 (17–56)	35.2 (16–56)	0.817
Female sex, n (%)	333 (63.1)	1614 (63.0)	0.993	896 (57.5)	4393 (57.6)	0.996
Socioeconomic status, n (%)			0.955			0.985
Low	139 (26.3)	660 (25.8)		400 (25.7)	1956 (25.6)	
Intermediate	321 (60.8)	1574 (61.5)		931 (59.8)	4556 (59.7)	
High	68 (12.9)	326 (12.7)		226 (14.5)	1121 (14.7)	
Ethnicity, n (%)			0.926			0.853
Arab	117 (22.2)	572 (22.3)		329 (21.1)	1629 (21.3)	
Jewish	411 (77.8)	1988 (77.7)		1228 (78.9)	6004 (78.7)	
	Comorbidities					
Body-mass-index (kg/m^2^), mean ± SD	28.1 ± 6	27.8 ± 6	0.553	26.2 ± 6	26.6 ± 9	0.050
Smoking ever, n (%)	187 (35.4)	838 (32.7)	0.233	429 (27.6)	2241 (29.4)	0.152
Cancer diagnosis, n (%)	85 (16.1)	304 (11.9)	0.009	159 (10.2)	535 (7.0)	<0.001
Cancer prior to index date n (%)	42 (8.0)	166 (6.5)	0.216	72 (4.6)	304 (4.0)	0.261
Age at cancer diagnosis, mean ± SD	53.3 ± 19	57.7 ± 14	0.160	52.8 ± 16	52.7 ± 18	0.946
Cancer post index date, n (%)	46 (8.7)	153 (6.0)	0.025	91 (5.8)	252 (3.3)	<0.001
Age at cancer, mean ± SD	57.9 ± 16	65.9 ± 14	0.004	55.6 ± 16	60.4 ± 14	0.011

Abbreviations: SD, standard deviation; IQR, interquartile range.

**Table 2:** Incidence of site-specific malignancies in polymyositis and dermatomyositis patients.

	Cancer Site ^a^	PM/DM, *n* (%)	Controls, *n* (%)	HR_age-and-sex_	95% CI	*p*
PM	All Cancers ^d^	46 (8.7)	153 (6.0)	1.50	1.06–2.11	0.020
	Solid Cancers ^b^	76 (14.4)	286 (11.2)	1.50	1.05–2.14	0.025
	Hematologic Cancers ^c^	4 (0.8)	13 (0.5)	1.63	0.53–5.07	0.395
	CNS	2 (0.4)	2 (0.1)	2.25	0.20–24.88	0.508
	Thyroid	3 (0.6)	8 (0.3)	1.40	0.29–6.72	0.677
	Breast	10 (1.9)	35 (1.4)	1.58	0.77–3.24	0.208
	Colorectal	5 (0.9)	20 (0.8)	0.96	0.33–2.83	0.946
	Kidney	3 (0.6)	4 (0.2)	3.28	0.55–19.68	0.194
	Bladder	2 (0.4)	10 (0.4)	1.22	0.26–5.76	0.800
	Prostate	6 (1.1)	14 (0.5)	2.42	0.91–6.46	0.077
	Uterus	4 (0.8)	7 (0.3)	1.21	0.13–10.82	0.865
	Melanoma	4 (0.8)	23 (0.9)	0.43	0.05–3.31	0.416
	Lung	1(0.2)	6 (0.2)	1.04	0.18–5.70	0.96
	Non-Hodgkin’s Lymphoma	3 (0.6)	7 (0.3)	2.09	0.54–8.08	0.286
DM	All Cancers ^d^	91 (5.8)	252 (3.3)	1.89	1.47–2.41	<0.001
	Solid Cancers ^b^	78 (5.0)	231 (3.0)	1.73	1.33–2.25	<0.001
	Hematologic Cancers ^c^	13 (0.8)	24 (0.3)	3.26	1.62–6.55	<0.001
	Oropharyngeal	2 (0.1)	3 (0.0)	4.99	0.70–35.45	0.108
	Larynx	2 (0.1)	2 (0.0)	10.16	0.92–112.04	0.058
	Thyroid	2 (0.1)	8 (0.1)	0.63	0.80–5.06	0.666
	Breast	17 (1.1)	49 (0.6)	1.86	1.07–3.25	0.028
	Colorectal	7 (0.4)	30 (0.4)	0.86	0.33–2.22	0.757
	Bladder	2 (0.1)	17 (0.2)	0.58	0.13–2.50	0.464
	Prostate	4 (0.3)	21 (0.3)	1.05	0.35–3.09	0.935
	Uterus	4 (0.3)	13 (0.2)	1.84	0.59–5.79	0.295
	Sarcoma	2 (0.1)	5 (0.1)	2.49	0.46–13.59	0.292
	Melanoma	3 (0.2)	17 (0.2)	0.99	0.29–3.43	0.990
	Lung	0	4 (0.1)	0.26	0.02–2.84	0.267
	Acute Leukemia	2 (0.1)	1 (0.0)	9.97	0.90–109.92	0.307
	Chronic Leukemia	6 (0.4)	7 (0.1)	5.02	1.62–15.56	0.005
	Hodgkin’s Lymphoma	2 (0.1)	1 (0.0)	9.89	0.90–109.09	0.061
	Non-Hodgkin’s Lymphoma	5 (0.3)	14 (0.2)	2.28	0.79–6.58	0.125

Abbreviations: CI, confidence interval; CNS, central nervous system; DM, dermatomyositis; PM, polymyositis; HR, hazard ratio. ^a^ only malignancies with more than one incident case during follow-up are presented. ^b^ includes the following malignancies: CNS, oropharyngeal, larynx, thyroid, lung, breast, uterus, ovaries, cervix, genital, kidney, bladder, prostate, esophagus, gastric, colorectal, liver and bile duct, pancreas, sarcoma and soft tissue, bone, other sites, unknown primary, and melanoma; ^c^ includes the following malignancies: acute leukemia, chronic leukemia, Hodgkin’s lymphoma, non-Hodgkin’s lymphoma, multiple myeloma, and myelodysplastic syndrome; ^d^ includes both solid and hematologic malignancies.

**Table 3:** Predictors of solid and hematologic malignancies within the polymyositis and dermatomyositis cohort. Age and sex adjusted logistic regression analysis.

	Solid Cancers (*n* = 121) ^a^			Hematologic Cancers (*n* = 17) ^b^		
	% ^c^ With Solid Cancer	OR_age-sex_ (95%CI)	*p*	% With Hematologic Cancer	OR_age-sex_ (95%CI)	*p*
Demographics						
Age at diagnosis, (5 years increment)	51.6 ± 16	1.14 (1.09–1.89)	<0.001	52.2 ± 16	1.14 (1.01–1.28)	0.032
Obesity	5.7%	0.70 (0.45–1.11)	0.130	0.4%	0.37 (0.08–1.64)	0.191
Female sex	6.8%	1.45 (0.97–2.17)	0.068	0.7%	0.54 (0.21–1.42)	0.213
Arab ethnicity	4.0%	0.85 (0.50–1.44)	0.540	0.2%	0.29 (0.04–2.25)	0.237
Low socioeconomic status	4.3%	0.82 (0.51–1.32)	0.413	0.6%	0.73 (0.21–2.61)	0.630
Inflammation/Necrosis Markers						
CPK (50 U/L increment)	1562 ± 3693	1.00 (0.99–1.00)	0.437	1547 ± 2868	1.00 (0.99–1.01)	0.887
CRP (5 mg/L increment)	15.3 ± 38	1.01 (0.99–1.04)	0.315	15.2 ± 20	1.01 (0.95–1.07)	0.751
Autoantibodies						
Myositis Specific						
Anti-Jo1	8.2%	1.40 (0.96–2.06)	0.083	1.7%	4.31 (1.45–12.80)	0.009
Anti-Mi2	11.8%	2.09 (1.22–3.58)	0.007	2.6%	3.83 (1.22–12.03)	0.021
Anti-Nuclear						
Any	8.1%	2.37 (1.44–3.91)	<0.001	1.2%	2.87 (0.77–10.76)	0.117
Anti-RNP/Sm	9.0%	1.70 (1.16–2.50)	0.007	1.4%	2.61 (0.95–7.18)	0.063
Anti-Ro/La	9.0%	1.79 (1.22–2.64)	0.003	1.5%	3.02 (1.06–8.57)	0.038
Anti-Scl-70	8.2%	1.60 (1.29–1.99)	<0.001	1.5%	2.70 (0.99–7.39)	0.053
Anti-DNA	8.6%	1.97 (1.31–2.99)	<0.001	1.5%	5.31 (1.44–19.55)	0.012
Anti-Phospholipid						
Any	10.9%	2.28 (1.55–3.35)	<0.001	1.2%	1.70 (0.61–4.75)	0.312
Anti-B2GP	10.8%	2.03 (1.35–3.05)	<0.001	1.1%	1.44 (0.46–4.53)	0.536
Anti-cardiolipin	11.0%	2.29 (1.55–3.37)	<0.001	1.3%	1.90 (0.68–5.30)	0.221
LAC	12.2%	2.29 (1.46–3.59)	<0.001	1.3%	1.71 (0.48–6.11)	0.410
Double positive	10.0%	2.02 (1.20–3.40)	0.008	1.4%	1.90 (0.51–6.98)	0.335
Triple positive	12.4%	2.70 (1.60–4.56)	<0.001	1.1%	1.70 (0.36–7.98)	0.504

Abbreviations: CI, confidence interval; CPK, creatinine phosphate kinase; CRP, C-reactive protein; LAC, lupus anticoagulant; OR, odds ratio. ^a^ includes the following malignancies: CNS, oropharyngeal, larynx, thyroid, lung, breast, uterus, ovaries, cervix, genital, kidney, bladder, prostate, esophagus, gastric, colorectal, liver and bile duct, pancreas, sarcoma and soft tissue, bone, other sites, unknown primary, and melanoma. ^b^ includes the following malignancies: acute leukemia, chronic leukemia, Hodgkin’s lymphoma, non-Hodgkin’s lymphoma, multiple myeloma, and myelodysplastic syndrome. ^c^ mean ± SD for continuous variables.

## Data Availability

The datasets presented in this article are not readily available due to the privacy policy of CHS.
